# Completing a Sustained Attention Task Is Associated With Decreased Distractibility and Increased Task Performance Among Adolescents With Low Levels of Media Multitasking

**DOI:** 10.3389/fpsyg.2021.804931

**Published:** 2022-02-07

**Authors:** John Brand, Reina Kato Lansigan, Natalie Thomas, Jennifer Emond, Diane Gilbert-Diamond

**Affiliations:** ^1^Department of Epidemiology, Geisel School of Medicine, Dartmouth College, Hanover, NH, United States; ^2^Norris Cotton Cancer Center, Geisel School of Medicine, Dartmouth College, Hanover, NH, United States; ^3^Department of Biomedical Data Sciences, Geisel School of Medicine, Dartmouth College, Hanover, NH, United States; ^4^Department of Medicine, Geisel School of Medicine, Dartmouth College, Hanover, NH, United States; ^5^Department of Pediatrics, Geisel School of Medicine, Dartmouth College, Hanover, NH, United States

**Keywords:** media multitasking, attention, adolescents, distraction, cognition

## Abstract

**Objective:**

To assess distracted attention and performance on a computer task following completion of a sustained attention and acute media multitasking task among adolescents with varying self-reported usual media multitasking.

**Methods:**

Ninety-six 13- to 17-year-olds played the video game Tetris following completion of a Go/No-go paradigm to measure sustained attention in the presence of distractors, an acute media multitasking, or a passive viewing condition. Adolescents completed the conditions on separate visits in randomized order. Sustained attention was measured within the Go/No-go task by measuring errors of omission. Distracted attention while playing the Tetris task was measured by computing eye tracking measures of attention (first fixation duration, cumulative fixation duration) to irrelevant distractor images that bordered the Tetris game. Participants also self-reported their daily media multitasking.

**Results:**

The Go/No-go task revealed important qualitative differences in sustained attention among low and high usual media multitaskers. There was a uniform improvement in sustained attention among low usual media multitaskers, demonstrated by a consistent linear decrease in omission errors (β = −0.01; *P* < 0.05). Among high usual media multitaskers, there was initially a decrease in sustained attention (β = −0.01; *P* = 0.05) followed by an increase (β = 0.16; *P* < 0.001). Completing the Go/No-go task also statistically significantly reduced distractibility and increased performance while playing Tetris compared to the passive viewing condition, but only among those with low usual media multitasking (all *P*s ≤ 0.05). There was a non-statistically significant trend that completing the acute media multitask increased subsequent distractibility and performance while playing Tetris among high media multitaskers.

**Conclusion:**

In this sample of adolescents, practicing a sustained attention task reduces distractibility and improves task performance among those who have low levels of usual media multitasking.

## Introduction

### Media Multitasking

Media multitasking—simultaneously attending to multiple forms of media—is ubiquitous among adolescents. From 1999 to 2009 adolescents nearly doubled their time spent media multitasking, from 16 to 29% of the time that they were using media (Rideout, 2013). The most recent data published in 2018 shows no signs of this trend slowing. Among adolescents between the ages of 13- and 17-years, 95% report having access to a cell phone and 45% state that they are online on a near constant basis (Pew Research Center, 2018).

### Sustained Attention and Media Multitasking

The rise in media multitasking has led to research exploring its relationship with the cognitive processes that underlie the ability to multitask (Alzahabi and Becker, 2013; Minear et al., 2013). Of particular focus has been the effect of media multitasking on sustained attention. Sustained attention can be divided into vigilance (detecting the appearance of a stimulus) and concentration (focusing on a stimulus or activity, while filtering out irrelevant distractors) (Sarter et al., 2001). Sustained attention is critical in performing tasks that require ignoring distractions and inhibiting attentional shifts to irrelevant stimuli. Deficits in sustained attention may have a disproportionate effect on people who engage in high levels of media multitasking (May and Elder, 2018). Previous research suggests that those with self-reported high levels of daily media multitasking have a reduced ability to ignore irrelevant information and increased distractibility (Ophir et al., 2009), as well as self-reported sustained attention problems, such as lapses of attention and attention-related errors (Ralph et al., 2014).

While previous research has shown that self-report measures of sustained attention relate to media multitasking, mixed results are found when sustained attention is measured using attentional paradigms in the laboratory. A single study showed that self-reported high levels of media multitasking were associated with deficits on one out of a battery of three sustained attention tasks (Ralph et al., 2014), whereas another study showed no association (Jun et al., 2018). These contradictory findings between self-report and performance-based measures of sustained attention have been taken to suggest that high media multitaskers may have increased failures of sustained attention in everyday life—as indexed by self-report measures—because they surround themselves more with distractions. In the absence of distractors, high media multitaskers can sustain their attention on a task in a laboratory setting (Ralph et al., 2014).

Media multitasking may also lead to carry-over effects. Carry-over effects occur when completing one task enhances or undermines performance on a subsequent task. Engaging in multiple tasks simultaneously drains attentional resources, resulting in deficits in cognitive control related to the ability to focus attention and filter irrelevant information (Miller and Cohen, 2001; Ophir et al., 2009; van der Schuur et al., 2015). These deficits may reduce performance on subsequent non-multitasking tasks after engaging in acute media multitasking (Courage et al., 2015). Conversely, practicing a sustained attention task may result in positive carry-over effects to reduce distractibility to improve subsequent task performance. Carry-over effects may be most pronounced among people with high usual media multitasking because of their documented propensity toward distractibility. No study has explored whether practicing a sustained attention task will result in carry-over effects while considering usual media multitasking. The present work aims to address this gap.

### Measuring Sustained Attention in the Presence of Distraction

The Go/No-go paradigm (Manly et al., 1999; Benikos et al., 2013) can be used to measure sustained attention (O’Connell et al., 2009). In these paradigms, participants are asked to respond (typically *via* a keyboard press) to a specific cue (a go stimulus) and withhold a response to a different cue (a no-go stimulus). Errors of omission occur when participants fail to respond to the presence of a stimulus (non-response on go-trials) and are thought to represent lapses in sustained attention. The overall error of omission percentage may be used as a measure of global sustained attention, where a higher percentage is evidence of low sustained attention. Increases and decreases in sustained attention may also be observed during the task by computing a moving average of the omission errors. When completed as part of a dual task, a decrease in the omission error moving average may represent an improvement in sustained attention attributed to an ability to ignore the competing task. Conversely, an increase in the moving average may represent a decrease in sustained attention attributed to difficulty in ignoring distractors.

### Measuring Distractibility

Distractibility during a primary task may be measured by monitoring eye movements (Folkvord et al., 2015; Brand et al., 2020). In these tasks, participants play a primary game, while a task irrelevant image is presented. The amount of distractibility may be measured by monitoring two eye movement measures to the distractor stimuli: first fixation duration and cumulative fixation duration. Within this context, first fixation duration is defined as the length of the first fixation to the distractor and represents the strength of an irrelevant stimulus to initially distract one’s attention from the primary task. It is thought to be driven by bottom-up processes, involving an automatic response to look at the stimulus because of its properties. Conversely, cumulative fixation duration is defined as the total amount of time a person fixates the distractors and represents the strength of a stimulus to distract one’s attention over time. It is defined as the total amount of time a person fixates the distractors and is thought to be driven by top-down processes, where the amount of time a person spends fixating the stimuli is related to their goals or how much they like the item.

### The Present Work

In the work that follows, we ask participants to play the video game Tetris to measure change from baseline measures of distractibility and performance, following a Go/No-go task designed to measure sustained attention. Critically, we asked adolescents to complete the Go/No-task in the presence of a distracting task to increase its ecological validity. We choose the game Tetris to measure distractibility and performance because it requires concentration and may reflect real world tasks that requires cognitive resources to perform well. Tetris can also be used to mimic advergames, popular online games for adolescents that require participants to play a video game in the presence of task irrelevant images, such as advertisements (van Berlo et al., 2020). Mimicking advergames may provide an ecologically valid way to measure how distracted participants are by measuring the amount of attention they give to task irrelevant images.

We hypothesized that practicing a sustained attention task would increase sustained attention, and that the magnitude would be greatest for adolescents who reported high levels of usual media multitasking. We further hypothesized that the practicing sustained attention would result in a decrease in distractibility and an increase in Tetris performance from baseline levels among high, but not low usual media multitaskers. Additionally, we collected adolescent demographics related to race, age, income, and education as potential covariates. These characteristics are robust predictors of media use, but their associations with media multitasking are unknown (Ulla, 2006).

## Materials and Methods

### Study Design

Ninety-six adolescents aged 13- to 17-years were recruited from the community using fliers, community listservs, social media, and community events. Relevant eligibility criteria included English fluency and absence of health conditions or medication use that may impact attention span, and willingness to participate in three 1.5-h study appointments. At each visit, adolescents completed the sustained attention or passive viewing baseline condition, randomized by laboratory visit. Immediately after, adolescents played the video game Tetris to measure task performance and measure the amount of attention that they gave to distractor images using eye tracking. Adolescents also reported on their usual media multitasking and demographics. In a different visit, we also asked adolescents to complete an acute multitask manipulation prior to completing the Tetris game. The purpose of this condition was to test a secondary hypothesis that completing a dual task designed to mimic media multitasking resulted in negative carry-over effects; that is, an increase in distractibility and a decrease in task performance from baseline measures. All study procedures were approved by The Committee for the Protection of Human Subjects at Dartmouth College. Adolescents provided assent, and parents provided consent.

### Stimuli and Apparatus

Experiment builder software was used to display the stimuli and control all timing and response operations. Stimuli were displayed on a 22-inch Elo 2201L monitor (Elo Touch Solutions, Knoxville, TN, United States) at a screen resolution of 1,920 × 1,080 and refresh rate of 60 Hz. There were three conditions: (1) acute media multitask (2) sustained attention, and (3) a passive viewing baseline condition. For all conditions, the screen was divided down the center. The left side of the screen presented yellow and blue dots that measured 4.0° degrees of visual angle when viewed at 60 cm. The location of the dots was randomized to appear within the boundaries of the left side. The right side of the screen contained an image of a mobile phone that displayed messages to the participant using an automated messaging bot.

### Conditions

#### Sustained Attention Condition

A modified Go/No-go task was used to measure sustained attention in the presence of a distracting task. For the task, a blue or yellow dot briefly appeared for between 20 and 1,500 msec and then disappeared. Participants were instructed to respond to the blue dot by pressing the space bar as quickly as possible (Go trials) and were told not to respond when presented with a yellow dot (No-go trials). At random intervals between 2,000 and 5,000 msec the messaging bot would ding and send participants a text message. The text message included questions about random facts; for example, how many teeth does an alligator have? Participants were instructed to ignore the messages and focus on the Go/No-go task. Participants were asked to ignore the competing text messages to contrast to acute multitask condition where they were asked to respond. Participants completed 100 Go trials and 100 No-go trials randomized on a trial-to-trial basis. There was a total of 60 messages sent to participants.

#### Acute Media Multitask Condition

Participants were asked to monitor and engage with two different tasks simultaneously. On the left side of the screen, a blue dot would briefly appear and then disappear (between 20 and 1,500 msec) and participants were instructed to press the space bar as quickly as possible whenever the blue dot appeared. On the right side of the screen, a messaging bot would ding and send participants a text message randomly every 2,000–5,000 msec. The questions were identical to those from the sustained attention task. Unlike the sustained attention condition, participants were asked to attend to and type answers to the text messages as possible. Participants were instructed to guess if they did not know the answer. Participants completed 100 trials and received a total of 60 messages.

#### Passive Viewing Baseline Condition

In the passive viewing condition, participants were asked to passively watch a video simulation of the media multitask condition with no specific instructions of where to look.

### Tetris

Participants were asked to play the video game Tetris while having their eye movements tracked. PyGames was used to create the stimuli and PyGaze software (Dalmaijer et al., 2014) was used to synchronize the eye tracker with the stimulus presentation. Binocular eye movements were recorded at 1,000 Hz using the Eyelink 1000 (SR research, Mississauga, ON, Canada). The Tetris game was presented inside a 768 × 1,366 rectangle presented in the middle of the screen. The game was bordered on the left and right by two gray rectangles, measuring 768 × 200 pixels. Distractor images were displayed in the center of the rectangles, measuring 200 × 200 pixels. One image was a picture of a food and the other was a picture of animal. The food and animal images were taken from the food-pics database (Blechert et al., 2014). The location of the images was randomized, and each image was replaced after 20 s. Total game play time was 5 min.

### Usual Media Multitasking

Participants reported on their usual media multitasking using the short form media multitasking index (Baumgartner et al., 2017). This index asks about media multitasking with other print and digital media during four primary activities: (1) watching TV or movies, (2) playing video games, (3) reading books or magazines (not assigned for school), and (4) doing homework. For each activity, participants reported the frequency with which they multitasked by engaging in the other activities by using a 5-point likert scale (i.e., 0 = *Never*, 1 = *Rarely*, 2 = *Sometimes*, 3 = *Often*, and 4 = *Always*). A usual media multitasking score was computed by taking the average of the Likert response. The score ranges from 0 to 4 with a higher score indicative of higher self-reported usual media multitasking.

### Child, Parent, and Household Characteristics

Parents provided their child’s age, sex, race, and ethnicity. Parents also reported their household income and their highest educational level completed.

## Dependent Variables

### Sustained Attention Performance

Sustained attention on the Go/No-go task was measured by computing omission errors, defined as when a participant failed to press the space bar within 2,000 msec of onset of a blue dot. An overall performance score was calculated for each participant as the percent of omission errors made. To assess whether sustained attention increased or decreased during the task, the 25-trial omission moving average was computed.

### Distractibility

The amount of attention given to distractor images while playing the video game Tetris was calculated for each participant after they completed each experimental condition. Areas of interest were created around distractor images, defined as the area of the image, 200 × 200 pixels. First fixation duration was measured as the average amount of time an adolescent spent fixating a distractor the first time that they looked at it. Cumulative fixation duration was calculated as the total amount of time participants spent fixating distractors, summed over all fixations. For both measures, a fixation duration was defined as any stationary period lasting at least 100 msec (Manor and Gordon, 2003).

### Tetris Performance

Performance on the Tetris task was evaluated by computing an overall Tetris score for each participant in each condition. The objective of Tetris is to position falling blocks of different shapes to complete as many horizontal lines as possible. For every line completed, participants are awarded a single point. In our game, a participant’s total Tetris score was the sum of their accumulated points over the 5 min of game play.

## Statistical Analysis

All analyses were conducted using the R language and environment for statistical computing (RStudio Team, 2020). Our original sample size was calculated using Cohen’s power calculation for regression. Setting alpha to *P* < 0.05, two-tailed, we calculated 80% power to detect a moderate effect size (*f*^2^ = 0.15) for main effects assuming a sample size of 107. However, we were forced to terminate the study early because the onset of the COVID-19 pandemic suspended in person laboratory experiments. Thus, our final sample size was 96 adolescents. Of the adolescents enrolled, one dropped out after visit one, two requested that their data not be used and a further seven were excluded because of poor eye-tracking calibration in at least one of the visits. Thus, there were a total of 86 participants with complete data who were included in the final analyses. For all models, we used a statistical significance threshold of *P* < 0.05 when evaluating main effects and a threshold of *P* < 0.10 when evaluating interactions.

### Child, Parent, and Household Characteristics

Distributions for each eye-tracking measure of distractibility, as well as usual media multitasking scores were compared across child, parent and household characteristics using unadjusted linear regression.

### Sustained Attention

The raw data for 25-trial omission error was plotted against trial number and visually inspected for linearity. Then, unadjusted mixed effect regression, nested within participant, was used to assess increases, or decreases in sustained attention.

### Effect of Sustained Attention Carry-Over Effects on Distractibility and Tetris Performance

We used unadjusted mixed effect regressions, nested within participant, to assess distractibility or Tetris performance metric by condition. Condition was coded as ordinal (1 = sustained attention; 0 = passive viewing baseline) as the predictor and first fixation duration, cumulative fixation duration, or Tetris raw values as the outcome.

### Effect Modification of Sustained Attention by Usual Media Multitasking

Overall errors of omission were compared between low and high usual media multitaskers (dichotomized at the median) using an independent samples *t*-test, two-tailed. The raw 25-trial omission error was plotted against trial number for low and high usual media multitaskers (median split) separately and visually inspected for linearity. If the data suggested a non-linear trend, we computed a mixed effects spline regression, specifying a single knot, predicting the 25-trial moving average from trial number. We then compared the resulting *R*-squared value to that computed from a mixed effects linear regression, nested within participants. In the case of a linear trend, we only fit a mixed effects linear regression, nested within participants, predicting the 25-trial omission moving average from trial number.

### Associations Between Usual Media Multitasking and Distractibility and Tetris Performance

We investigated the associations between baseline distractibility, baseline Tetris performance, and usual media multitasking. After inspecting for linearity, separate unadjusted linear regressions were used to predict first fixation duration, cumulative fixation duration, and Tetris scores in the passive viewing baseline condition from usual media multitasking coded as continuous.

### Effect Modification by Usual Media Multitasking on the Effect of Sustained Attention Carry-Over Effects on Distractibility and Tetris Performance

To investigate how usual media multitasking modified the effect of sustained attention carry-over effects on distractibility and Tetris performance, we dichotomized participants into low and high usual media multitaskers at the median and fit separate unadjusted mixed effects models, nested within participants. First fixation duration, cumulative fixation duration, and Tetris scores were predicted from models that included condition coded as binary (0 = passive viewing baseline; 1 = sustained attention). We then constructed additional models that also included a multiplicative interaction between condition coded as binary (0 = passive viewing baseline; 1 = sustained attention) and usual media multitasking coded as continuous and conducted a Wald test of significance on the interaction term.

### Effect of Acute Media Multitasking on Distractibility and Tetris Performance

We repeated analyses for the acute media multitask vs. passive viewing baseline to assess how the acute media multitask affected distractibility and Tetris performance metrics and whether the associations differed by usual media multitasking.

## Results

### Child, Parent, and Household Characteristics

Table 1 shows the distribution of attention metrics, as well as usual media multitasking across child, parent, and household characteristics. About half of the children were male (54.6%) and the vast majority were between 13 and 15 years of age (80.2%) and predominately white, non-Hispanic (82.5%). Furthermore, most children were recruited from households with an annual income greater than $65,000 (88.3%), and a mother’s educations level of at least an associate or bachelor’s degree (94.1%). There were no significant associations between participant characteristics and the attentional or usual multi-tasking metrics.

**TABLE 1:** Distribution of attention metrics and usual media multitasking scores across child, parent, and household characteristics.

		First fixation duration (msec)	Cumulative fixation duration (msec)	Usual media multitasking
	N	Mean	Mean	Mean^*^
**Overall**	86			
**Child characteristics**				
Age, years				
13	25	449.92	19604.33	2.24
14	20	410.90	10369.10	2.18
15	24	551.58	25625.33	2.59
16	14	370.14	15075.71	2.19
17	3	395.33	8692.00	3.41
β (95% CI)		−9.53 (−81.48, 62.41)	−184.18 (−4648.10, 4279.74)	−0.89 (−1.71, 0.92)
Sex				
Male	47	477.11	21201.40	2.35
Female	39	438.11	14041.16	2.36
β (95% CI)		−39.00 (−207.10, 129.09)	−7160.25 (−17481.37, 3160.88)	0.02 (−0.362, 0.402)
Race^&^				
White, non-Hispanic	71	499.50	14535.00	2.26
Other, non-Hispanic	12	438.58	18535.92	2.36
β (95% CI)		−54.73 (−285.25, 175.77)	4261.99 (−10779.25, 19303.22)	0.109 (−0.430, 0.649)
**Parent characteristics** ^&^				
Mother’s education level				
High school diploma	2	680.00	70847.00	2.43
Associate or Bachelor’s degree	69	496.83	17061.67	2.53
Graduate school	12	438.41	16848.87	2.32
β (95% CI)		−170.83 (−545.96, 204.30)	−38182.44 (−61314.51 −15050.37)	−0.12 (−0.77, 0.52)
**Household characteristics** ^&^				
Annual income				
$0−64, 999	4	350.00	5081.00	2.59
$65,000−144,999	64	452.66	17849.72	2.36
> $145,000	12	441.50	21256.50	2.25
β (95% CI)		64.70 (−240.58, 369.98)	11437.81 (−8230.13, 31105.74)	0.045 (−0.40, 0.49)

*Beta and 95% confidence intervals around the beta were calculated from linear regression models with the participant characteristic as the single exposure and either the attention metric or usual media multitasking as the continuous outcome. Age coded as continuous. Sex and race coded as binary. Mother’s education level coded as ordinal. 0 = some high school, 1 = High school graduate or GED; 2 = Associates degree, 3 = Bachelor’s degree, 4 = Graduate school (including medical and law school). Household income coded as ordinal: 0 = less than $25, 000, 1 = $25, 000–64,999, 2 = $65,000–144,999, 3 = $145, 000–224,999, 4 = 225,000 or more.*

*Usual media multitasking scores range from 0 to 4 with a higher score indicative of higher self-reported usual media multitasking.

*^&^3 participants did not report their race or parental education. 6 participants did not report their income.*

### Sustained Attention

The overall average mean omission error was 27.64 (SD = 8.67). For every increase in Trial number the 25-trial omission moving average decreased by −0.13 (*P* < 0.01), suggesting that sustained attention increased during the task.

### Effect of Sustained Attention Carry-Over Effects on Distractibility and Tetris Performance

Overall, the average Tetris score was 4.99 (SD = 3.65; min = 0; max = 15). When examining measures of distractibility and Tetris performance following sustained attention, there were qualitative differences in the hypothesized directions, although the results were not statistically significant. Compared to after the passive viewing baseline, the first fixation duration (β = −25.11; *P* = 0.64) and cumulative fixation duration (β = −674.9; *P* = 0.83) were both lower following completion of the sustained attention task, and Tetris scores were higher (β = 0.494; *P* = 0.36).

### Usual Media Multitasking and Sustained Attention Task Performance

**FIGURE 1:**
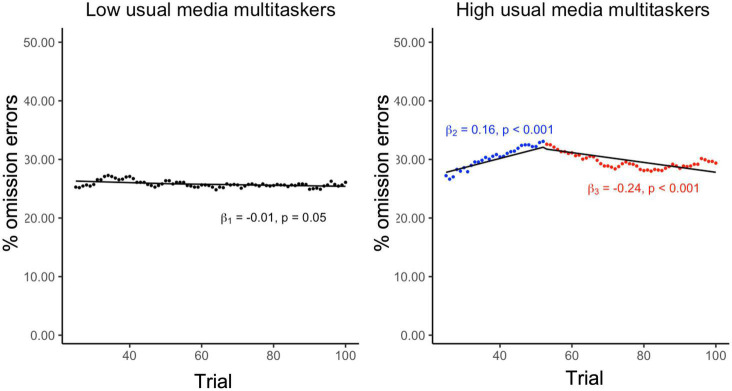
Sustained attention task performance presented as the 25-trial omission error rate by usual media multitasking dichotomized at the median. Regression lines plotted over the raw values. β1: For the sustained attention condition data, β from a mixed effect linear regression, nested within participant, for trial number predicting the 25-trial moving average among low usual media multitaskers. β_2_ and β_3_: For the sustained attention condition data, βs from a mixed effect spline regression, specifying a single knot and nested within participant, predicting the 25-trial moving average among high usual media multitaskers.

Overall, low usual media multitaskers had higher levels of sustained attention, measured as an overall lower level of omission errors when compared to high usual media multitaskers (0.30 vs. 0.34; *P* < 0.001). When tracking changes in sustained attention, there were qualitative differences between high and low usual media multitaskers. Visual inspection revealed a non-linear trend in the 25-trial omission moving average among high media multi-taskers and a linear trend in the 25-trial omission moving average among low media multitaskers (Figure 1). Among high usual media multitaskers, the 25-trial omission moving average was associated with a statistically significant increase over the first 52 trials (β = 0.16; *P* < 0.001) followed by statistically significant decrease (β = –0.24; *P* = 0.001) (Figure 1). The overall adjusted *R*^2^ for this model was 0.632, greater than the adjusted *R*^2^ value of 0.19 resulting from fitting a single linear trend. Conversely, among low usual media multitaskers, the 25-trial omission moving average was associated with a marginally statistically significant linear decrease (β = −0.01; *P* < 0.05), with an adjusted *R*^2^ value of 0.19.

### Baseline Associations Between Usual Media Multitasking, Attention to Distractors, and Tetris Performance

Usual media multitasking was not statistically significantly associated with any eye movement measures of attention to distractors after the passive viewing baseline condition, though the associations were in the expected directions. For each one unit increase in usual media multitasking, first fixation duration decreased by −21.67 (*P* = 0.66) and cumulative fixation duration decreased by −1401.00 (*P* = 0.64). Usual media multitasking was also not statistically significantly associated with Tetris performance. For each one unit increase in usual media multitasking, Tetris scores increased by 0.385 (*P* = 0.14).

### Effect Modification by Usual Media Multitasking on the Effect of Sustained Attention Carry-Over Effects on Distractibility and Tetris Performance

**FIGURE 2:**
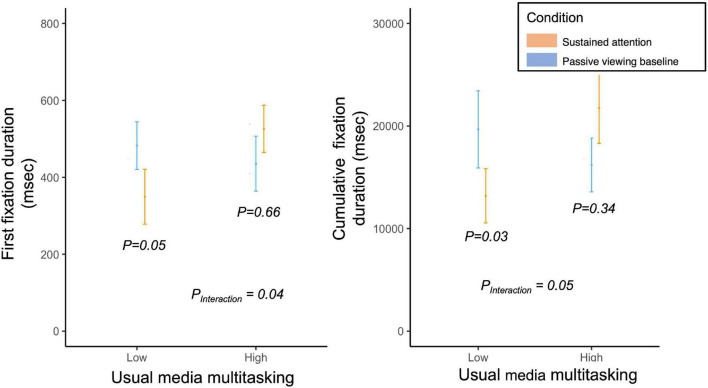
Eye tracking metrics of attention to distractor images while playing Tetris following completion of the sustained attention and passive viewing baseline conditions. Data stratified by usual median multitasking and presented as mean ± 1.96 × SEM. P_Interaction_: Mixed effect model, nested within participant, predicting first fixation duration or cumulative fixation duration from condition coded as binary (0 = control; 1 = sustained attention), usual media multitasking coded as continuous and a multiplicative interaction between the two.

When examining measures of distractibility following completion of the sustained attention vs. passive viewing baseline, there were differences between low and high usual media multitaskers (Figure 2). For those with low usual media multitasking, completing the sustained attention task resulted in a lower first fixation duration and cumulative fixation duration compared to baseline (*P* = 0.05 and *P* = 0.03, respectively); however, there was no sustained attention effect among those with high usual media multitasking. The test for interaction between the sustained attention vs. baseline and usual media multi-tasking modeled as a continuous variable was statistically significant (*P* = 0.04).

**FIGURE 3:**
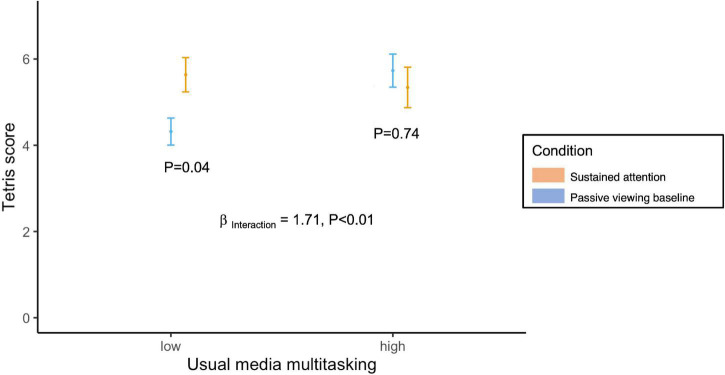
Tetris performance in the presence of distractors following completion of the sustained attention and passive viewing conditions by usual media multitasking (dichotomized at the median). Data presented as mean ± 1.96 × SEM. P_Interaction_: Mixed effect model, nested within participant, predicting Tetris score from condition coded as binary (0 = control; 1 = sustained attention), usual media multitasking coded as a continuous predictor, and a multiplicative interaction between the two.

There was a similar effect when examining the Tetris performance following completion of the sustained attention vs. baseline conditions (Figure 3). For those with low usual media multitasking Tetris scores were statistically significantly higher after completing the sustained attention task compared to baseline (*P* = 0.04); however, there was no sustained attention carry-over effect on Tetris scores among high usual media multitaskers. There was a statistically significant interaction between the sustained attention and usual media multitasking modeled as a continuous variable; (*P* < 0.01).

### Effect of Acute Media Multitasking on Distractibility and Tetris Performance

When examining measures of distractibility and Tetris performance following completion of the acute media multitask, there were qualitative differences in the hypothesized directions, although the results were not statistically significant. Compared to baseline, the first fixation duration (β = 35.93; *P* = 0.51) and cumulative fixation duration (β = 3657.9; *P* = 0.24) were both higher following completion of the acute media multitask, and Tetris scores were lower (β = −0.52; *P* = 0.34). There was no significant effect modification on either first fixation duration (β = 4.47; *P* = 0.97), cumulative fixation duration (β = −128.70; *P* = 0.98), or Tetris scores (β = 0.508; *P* = 0.60) following the acute media multitask condition by median multitasking.

## Discussion

In the present work, we measured sustained attention in the presence of a distracting task and possible carry-over effects among participants with varying degrees of media multitasking. Overall, sustained attention improved during the Go/No-go task among low and high usual media multitaskers, although there were important qualitative differences. There was a uniform improvement in sustained attention among low usual media multitaskers, demonstrated by a consistent linear decrease in omission errors. Among high usual media multitaskers, however, there was initially a decrease in sustained attention followed by an increase. There were also important differences when comparing Tetris performance. Distractibility was reduced and performance was increased, but only among low usual media multitaskers. Our findings add to the growing literature which support that the association between media multitasking and sustained attention may lead to measurable effects on real life tasks.

Providing partial support for our hypothesis, overall sustained attention levels were lower among participants with self-reported high usual media multitasking as indicated by a greater overall omission error rate. Nevertheless, and contrasting our predictions, sustained attention improved throughout the Go/No-go task regardless of self-reported media multitasking level. This suggests that to some extent sustained attention improved independent of media multitasking level, although potential carry-over effects were only present among low usual media multitaskers—demonstrated by decreased distractibility and increased Tetris performance. The lack of evidence for carry-over effects among high usual media multitaskers may be attributable to cognitive fatigue. Our sustained attention task was presented alongside the presence of a secondary distracting task, for which research suggests that high usual media multitaskers may have been more distracted (Ophir et al., 2009; Cain and Mitroff, 2011). We predicted that the sustained attention task may subvert the presence of distractors, and our results are consistent with this interpretation with an important caveat. Sustained attention among high media multitasker initially decreased before rebounding, suggesting an initial difficulty in filtering the distracting task. The additional cost of filtering among high usual multitaskers may have increased cognitive demand leading to increased fatigue during the Go/no-task that carried over to the Tetris game.

It may also be that high media multitaskers required a higher dose of sustained attention to see increased carry-over effects. While needing to be confirmed by future research, this suggests that practicing more intensive sustained attention tasks may help negate the negative effects of media multitasking. One potential candidate is mindfulness. Mindfulness interventions may be uniquely promising because the mechanisms associated with mindfulness are the opposite of the automatic, poorly filtered attention thought to underlie the negative effects of media multitasking (Gorman and Green, 2016). Mindfulness is the ability to focus and self-sustain mindful, conscious processing of task-relevant stimuli that would otherwise lead to habituation and subsequent distraction to extraneous stimuli. A short-term mindfulness intervention among a large sample of young adults showed that mindfulness training improved performance on a battery of attention tasks—including filtering and distractibility in both high and low media multitaskers, but this effect was greater for high media multitaskers (Gorman and Green, 2016).

The present work has the following limitations. We attempted to measure the effect of acute media multitasking on distraction and task performance by developing a novel acute media multitask. We found a non-statistically significant trend that completing this task increased subsequent distractibility and performance while playing Tetris. However, our ability to interpret this result is limited because of our inability to measure engagement with and performance on the task among our participants. We intended to assess these metrics using eye tracking technology. However, eye movement data was unreliable because of a high number of dropped samples as participants shifted their gaze from the screen to the keyboard to answer the messaging bot. Future research attempting to manipulate media multitasking should consider the most effective way to measure engagement and performance during a multitask to ensure robust data collection and adequate controls, such as measuring whether one task is more engaging than the other. Participants in our design also engaged in a specific form of multitasking involving the continuous switching of attention among multiple sources of information on the same device. Media multitasking may also involve not only switching attention between tasks, but also between devices. Dividing attention among tasks and multiple media forms simultaneously may place extra demands on the cognitive system and lead to greater multitasking effects. We also attempted to investigate participant demographics as possible covariates, but our sample was predominantly white, non-Hispanic and recruited from households with high income and parental education levels. Future work is needed to examine if associations are similar across various socioeconomic groups. Distractor images in our study were limited to food and animal images. Results may therefore not generalize to the image complexity and heterogeneity found in the environment.

The present work has the following strengths. To our knowledge, our study is the first to measure sustained attention in the presence of task to mimic a media task. It is also the first to suggest carry-over effects associated with practicing a sustained attention task in a controlled laboratory environment, showing that increases in sustained attention are associated with a decrease in baseline distractibility and video game performance. Future research should investigate carry-over effects on real world behaviors known to be affected by media multitasking, such as academic performance. Our work is also the first to investigate how usual media multitasking may modify carry-over effects on distractibility and task performance. Our finding of a significant effect modification may suggest that practicing sustained attention may help to promote positive carry-over effects, although future research is needed to determine if these effects would be most effective when tailored to an individual’s usual media multitasking level.

## Conclusion

Practicing a sustained attention task may lead to carry-over effects to reduce distractibility and improve task performance among those who have low levels of usual media multitasking. Future studies are needed to clarify whether carry-over effects associated with sustained attention may be efficacious in reducing distractibility and increasing task performance among high usual media multitaskers. Additional work is also needed to determine the effect of acute media multitasking on distractibility and task performance.

## Data Availability Statement

The raw data supporting the conclusions of this article will be made available by the authors, without undue reservation.

## Ethics Statement

The studies involving human participants were reviewed and approved by the Committee for the Protection of Human Subjects at Dartmouth College. Written informed consent to participate in this study was provided by the participants’ legal guardian/next of kin.

## Author Contributions

JB contributed to the conception and design of the study, performed the statistical analyses, and wrote the first draft of the manuscript. JE contributed to the conception of the study and performed the statistical analyses. RL and NT contributed to the conception and design of the study and wrote specific sections of the manuscript. DG-D contributed to the conception and design of the study and provided critical edits to the manuscript. All authors contributed to manuscript revision and read and approved the submitted version.

## Conflict of Interest

The authors declare that the research was conducted in the absence of any commercial or financial relationships that could be construed as a potential conflict of interest.

## Publisher’s Note

All claims expressed in this article are solely those of the authors and do not necessarily represent those of their affiliated organizations, or those of the publisher, the editors and the reviewers. Any product that may be evaluated in this article, or claim that may be made by its manufacturer, is not guaranteed or endorsed by the publisher.
